# Somatosensory Nerve Fibers Mediated Generation of De-qi in Manual Acupuncture and Local Moxibustion-Like Stimuli-Modulated Gastric Motility in Rats

**DOI:** 10.1155/2014/673239

**Published:** 2014-04-30

**Authors:** Yang-Shuai Su, Zhao-Kun Yang, Juan-Juan Xin, Wei He, Hong Shi, Xiao-Yu Wang, Ling Hu, Xiang-Hong Jing, Bing Zhu

**Affiliations:** Institute of Acupuncture and Moxibustion, China Academy of Chinese Medical Sciences, 16 Nanxiaojie, Dongzhimennei, Bejing 100700, China

## Abstract

The aim of this study was to reveal the somatosensory nerve fibers mediated generation of De-qi in manual acupuncture stimuli (MAS) and local moxibustion-like stimuli (LMS). The effects of strong and slight MAS, as well as 41°C, 43°C, and 45°C LMS at ST36 and CV12 on gastric motility were observed in rats. Gastric motility was continuously measured by an intrapyloric balloon, and the average amplitude, integral, and frequency of gastric motility during LMS were compared with those of background activity. Gastric motility was facilitated by MAS and LMS at ST36 and inhibited at CV12. The modulatory effects induced by strong MA with potent De-qi (needle grasp feeling) were markedly higher than those by slight MA with mild De-qi sensation (*P* < 0.05). The nociceptive 43°C and 45°C LMS, rather than nonnociceptive 41°C LMS, produced significant regulatory effects on gastric motility. Based on the afferent fibers activated in the present study, these results support the hypothesis that A**δ**- and C-afferent fibers were more likely to be involved in the generation of De-qi sensation.

## 1. Introduction

Manual acupuncture and moxibustion, as two traditional Chinese medicinal techniques, have been widely used to treat a wide range of diseases in clinical practice in China. During the past decades, the therapeutic effects of manual acupuncture and moxibustion on digestive disorders have been investigated and partially confirmed [1, 2]. Some regular responses of gastrointestinal tract contraction induced by acupuncture and moxibustion stimulation have been observed in previous studies [3, 4]. Additionally, it is suggested that acupuncture and moxibustion stimuli with different intensities are more likely to activate distinct afferent fibers to achieve their therapeutic effects [5, 6].

Among factors contributing to the effectiveness of manual acupuncture and moxibustion treatment, De-qi feeling is considered the most critical one [7]. For manual acupuncture stimulation, De-qi is elicited by rotating, lifting, and thrusting the inserted needle and perceived by the patient as “distention,” “heaviness,” or “soreness.” “Needle grasp” sensation perceived by acupuncturist during manual manipulation is also an important indicator of De-qi, which is also classically described as “like a fish biting on a fishing line.” Unlike manual acupuncture, De-qi sensation elicited by moxibustion stimulation is described as heat-sensitive moxibustion sensation, including penetrating heat, expanding heat, and transmitting heat [8]. Although De-qi sensations induced by manual acupuncture and moxibustion are different, both are closely related to the function of afferent terminals innervating muscles and connective tissues beneath acupoints [9].

Based on the possible correlation between intensity of stimulation, distinct afferent fibers, and generation of De-qi, we hypothesized that manual acupuncture or moxibustion stimuli with certain intensities could elicit De-qi feeling via activating specific afferent fibers. In order to reveal the correlation further, the effects of strong and slight manual acupuncture stimuli (MAS) as well as nonnociceptive (41°C) and nociceptive (43°C, 45°C) local moxibustion-like stimuli (LMS) on gastric motility in rats were investigated in the present study.

## 2. Materials and Methods

### 2.1. Animal Preparation

Male Sprague-Dawley (SD) rats (*n* = 36), weighing 250–300 g, were purchased from Institute of Animal, Academy of Chinese Medical Sciences. The animals were housed under a 12 h light/dark with free access to food and water. All animals were treated according to the Guide for Use and Care of Medical Laboratory Animals from the Ministry of Public Health of People's Republic of China.

### 2.2. Gastric Motility Recording

The animals were fasted overnight with free access to water. For anesthesia, 10% urethane (1.0–1.2 g/kg, via intraperitoneal route) was administered. Gastric motility was recorded by inserting a small balloon via an incision of duodenum into the pyloric area as described previously [10]. In order to detect the gastric contraction, 0.2-0.3 mL warm water was injected into the balloon to keep the basal pressure at about 100 mm H_2_O.

Changes in pressure of the balloon were measured continuously by a transducer and then input into a polygraph amplifier (NeuroLog, NL900D). The signal was captured online and analyzed offline using a data acquisition system (Power-Lab/4s, AD Instruments) and Chart 5.2 software. Gastric contraction was recorded as a control for at least 30 min before any stimulation. Responses induced by MAS or LMS were compared with the background activity in terms of average amplitude (the average difference between the cyclic maxima and minima in the selected cycles), integral (calculated as the sum of the data points multiplied by the sample interval), and frequency (per minute) of gastric contraction waves. Systemic blood pressure and heart rate were monitored by using Biopac data acquisition system (MP150, USA), and rectal temperature was kept constantly around 37°C by a feedback-controlled heating blanket (DC, USA).

### 2.3. Manual Acupuncture Stimuli (MAS) and Local Moxibustion-Like Stimuli (LMS) of CV12 and ST36

MAS and LMS were performed at ST36 (Zusanli) or CV12 (Zhongwan). ST36 is located 5 mm below the head of the fibula under the knee joint and 2 mm lateral to the anterior tubercle of the tibia. CV12 is located 4 cm below the processus xiphoideus, in the middle line of the abdomen. Rats were randomly divided into four groups: MAS + ST36 group, MAS + CV12 group, LMS + ST36 group, and LMS + CV12 group (*n* = 9, in each group). To minimize mutual interference, the noninvasive LMS was applied prior to MAS. Hair located around the acupoints was cut off to expose the local skin before LMS application. The LMS was performed by application of a heat generator (Physitemp Controller NTE-2A, Physitemp Instruments INC, USA) connected with a probe (2 cm in diameter) to avoid burning by real moxibustion. The contact area between the probe and skin (acupoints) is about 1 cm in diameter. The stimulation parameters of the instrument were set at 41°C, 43°C, and 45°C, respectively. When the temperature was stable, the LMS would be given by attaching the probe to the skin area (acupoints) for 180 s. Rats were allowed to stabilize for at least 30 min after the LMS.

When the gastric contraction wave recovers to control level, MAS with a needle of 0.3 mm in diameter was inserted about 5 mm into the skin and its underlying muscles at ST36 or CV12. The needle was rotated clockwise and anticlockwise for 60 s at 1 Hz and 2 Hz in slight MAS and strong MAS, respectively [11]. During each strong MAS, the “needle grasp” sensation can be perceived obviously by the acupuncturist, which is more stronger than that induced by slight MAS.

Both LMS and MAS were applied at ST36 or CV12 in an ascending order. The latter stimulus can only be applied when the gastric motility recovered to control level. The background gastric activity and gastric activity during and after LMS and MAS were recorded continuously, 60 s for each session.

### 2.4. Statistical Analysis

Changes in the average amplitude and integral were calculated according to (the value during stimulation − the value before stimulation) ÷ the value before stimulation × 100%. The data obtained before and after treatment in the same group or different group was compared statistically by a paired *t*-test or unpaired *t*-test. *P* < 0.05 was considered as a statistical significance. All data are expressed as mean ± SE.

## 3. Results

### 3.1. Facilitatory Effects on Gastric Motility Induced by Different LMS at ST36

**Figure 1:**
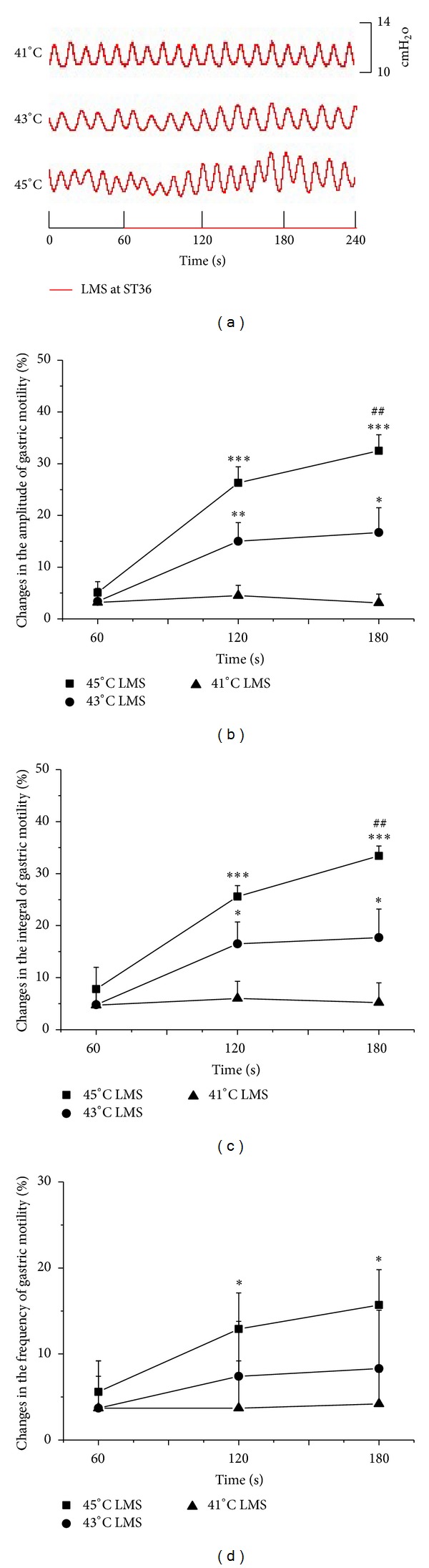
Gastric motility in response to LMS at ST36 with different intensities in rats. (a) Examples of the alterations of gastric contraction wave induced by different intensities of LMS at ST36. (b, c, and d) The changes of the amplitude, integral, and frequency of gastric motility induced by LMS at ST36 in total 180 s, respectively (*n* = 9; *P < 0.05, **P < 0.01, and ***P < 0.001, versus background activities; ^##^ *P* < 0.01, as compared with the facilitatory effects in the same time course of 43°C LMS at ST36).

LMS at ST36 induced facilitatory effects which were dependent on the intensity.  Figure 1(a) showed typical responses of gastric motility following LMS with three different temperature stimulations for 180 s. Figures 1(b), 1(c), and 1(d) summarized the responses obtained from all 9 tested rats. It should be noted that 41°C LMS had no significant impact on the amplitude and integral of gastric motility. In addition, both 43°C and 45°C at ST36 failed to produce any marked changes during the first 60 s of LMS. However, 43°C and 45°C LMS at ST36 elicited a significant enhancement on the amplitude and integral of gastric contraction in the last 120 s compared with the background activities (amplitude changes in the second and third 60 s: 43°C: 15.0 ± 3.6%, 16.7 ± 4.8%, *P* < 0.01, *P* < 0.05; 45°C: 26.3 ± 3.1%, 32.5 ± 3.1%, *P* < 0.001) (integral changes in the second and third 60 s: 43°C: 16.5 ± 4.2%, 17.7 ± 5.5%, *P* < 0.05; 45°C: 25.6 ± 2.1%, 33.4 ± 1.9%, *P* < 0.001). A 60-second latency was also observed before the emergence of the LMS-modulated gastric motility. In addition, the facilitatory effects induced by 45°C LMS at ST36 were significantly higher than those by 43°C LMS in terms of the amplitude and integral of gastric motility (*P* < 0.05).

Figure 1(d) illustrated the impact of LMS at ST36 on the frequency of gastric motility. 41°C and 43°C LMS failed to bring about any significant response, while 45°C LMS at ST36 induced significant enhancement on the frequency of gastric motility compared with the background activities, which also appeared in the last 120 s of stimulation (frequency changes in the second and third 60 s of 45°C LMS: 12.9 ± 4.2%, 15.7 ± 4.1%, *P* < 0.05).

### 3.2. Inhibitory Effects on Gastric Motility Induced by LMS at CV12

**Figure 2:**
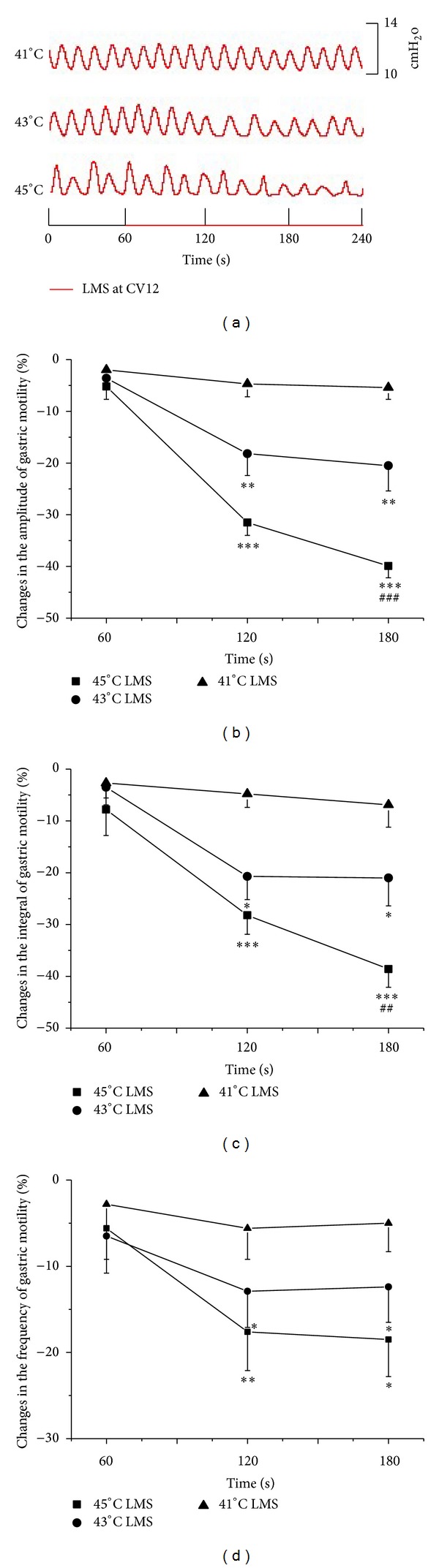
Gastric motility in response to LMS at CV12 with different intensities in rats. (a) Examples of the alterations of gastric contraction wave induced by different intensities of LMS at CV12. (b, c, and d) Changes of the amplitude, integral, and frequency of gastric motility induced by LMS at CV12 in total 180 s, respectively (*n* = 9; *P < 0.05, **P < 0.01, and ***P < 0.001, versus background activities; ^##^ *P* < 0.01 and ^###^ *P* < 0.001, as compared with the inhibitory effects in the same time course of 43°C LMS at CV12).

LSTS at CV12 induced inhibitory effects which were also dependent on the intensity.  Figure 2(a) showed typical responses of gastric motility following LMS with three different intensities for 180 s, and Figures 2(b), 2(c), and 2(d) summarized the responses obtained from all 9 tested rats. There was no significant change of gastric motility during 41°C LMS. During the second and third 60 s of 43°C and 45°C LMS, the gastric motility was markedly inhibited by CV12 (amplitude changes: 43°C: −18.2 ± 4.2%, −20.5 ± 4.9%, *P* < 0.01; 45°C: −31.5 ± 2.5%, −39.9 ± 2.3%, *P* < 0.001) (integral changes: 43°C: −20.7 ± 4.5%, −21.0 ± 5.4%, *P* < 0.05; 45°C: −28.2 ± 3.7%, −38.6 ± 3.5%, *P* < 0.001) (frequency changes: 43°C: −12.9 ± 4.2%, −12.4 ± 4.1%, *P* < 0.05; 45°C: −17.6 ± 4.5%, *P* < 0.01; −18.5 ± 4.3%, *P* < 0.05). Similarly, the inhibitory effects of 45°C LMS at ST36 on the amplitude and integral of gastric motility were significantly higher than those by 43°C LMS (*P* < 0.05). Besides the existence of the short latency, the temperature-specific (43°C) manner was quite obvious in the responses of gastric motility to LMS at ST36 and CV12.

### 3.3. Modulation on Gastric Motility Induced by Different MAS at ST36 or CV12

**Figure 3:**
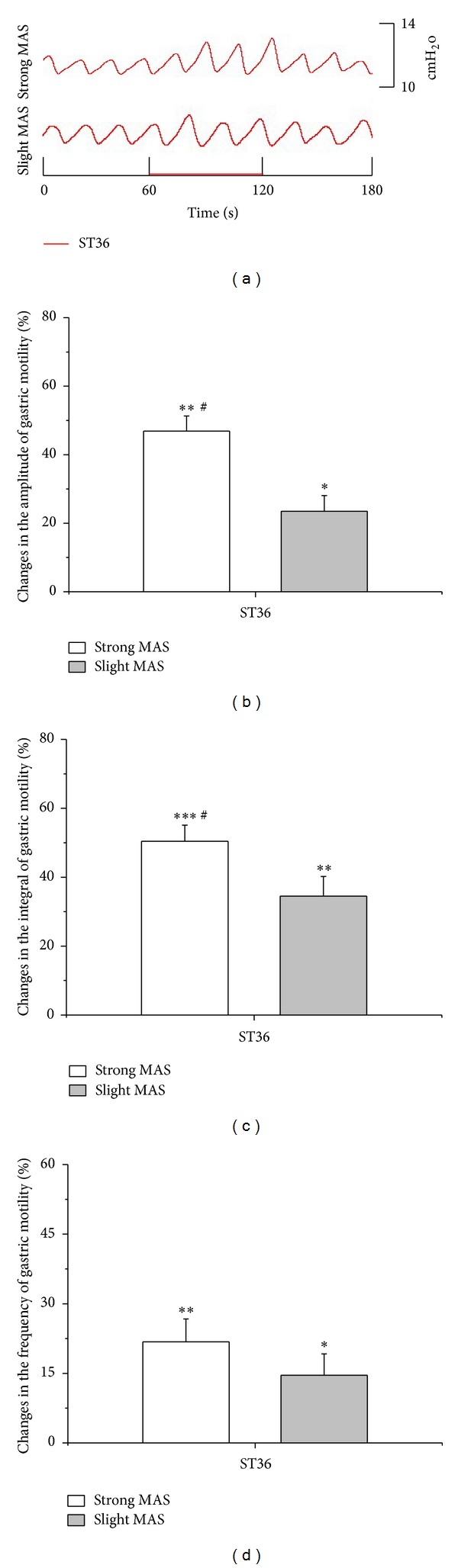
Gastric motility in response to MAS at ST36 with different intensities in rats. (a) Examples of the alterations of gastric contraction wave induced by different intensities of MAS at ST36. (b, c, and d) Changes of the amplitude, integral, and frequency of gastric motility induced by MAS at ST36 in 60 s, respectively (*n* = 9; *P < 0.05, **P < 0.01, and ***P < 0.001, versus background activities; ^#^ *P* < 0.05, as compared with the facilitatory effects of slight MAS at ST36).

**Figure 4:**
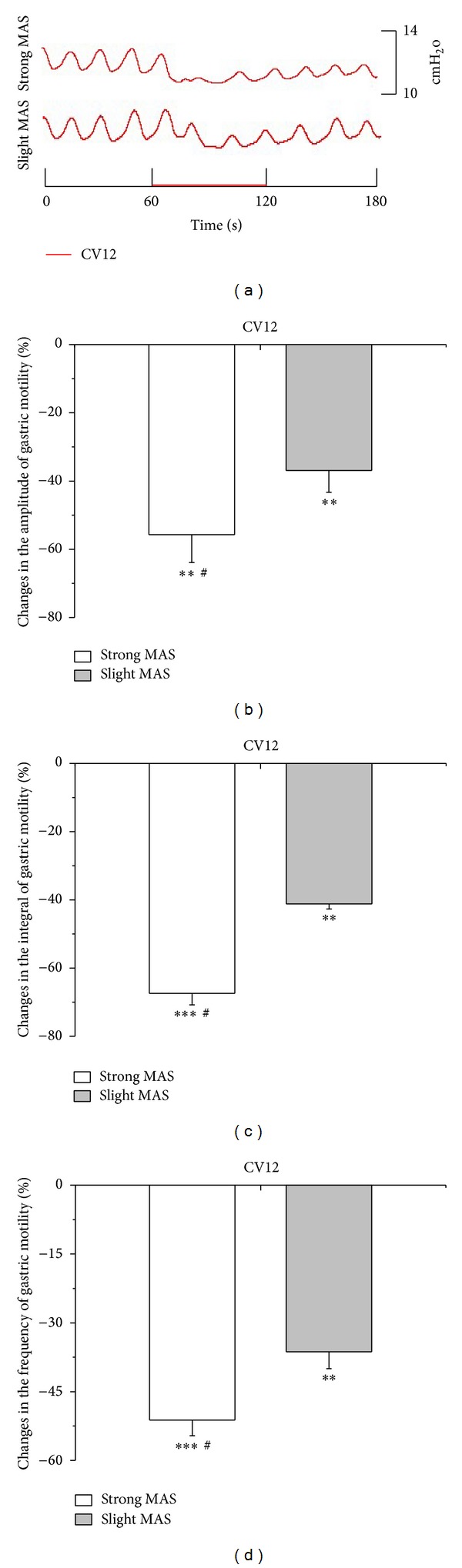
Gastric motility in response to MAS at CV12 with different intensities in rats. (a) Examples of the alterations of gastric contraction wave induced by different intensities of MAS at CV12. (b, c, and d) Changes of the amplitude, integral, and frequency of gastric motility induced by MAS at CV12 in 60 s, respectively (*n* = 9; **P < 0.01 and ***P < 0.001, versus background activities; ^#^ *P* < 0.05, as compared with the inhibitory effects of slight MAS at CV12).

**Figure 5:**
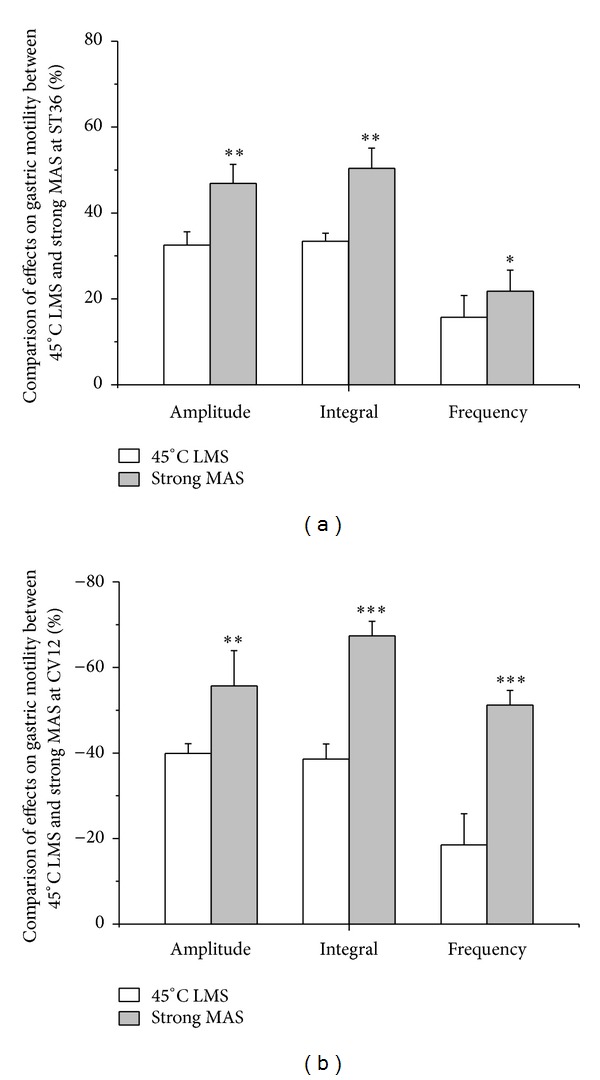
Comparison of the regulatory effects on gastric motility between the last 60 s of 45°C LMS and strong MAS. (a) Comparison of the facilitatory effects on gastric motility between the last 60 s of 45°C LMS and strong MAS at ST36. (b) Comparison of the inhibitory effects on gastric motility between the last 60 s of 45°C LMS and strong MAS at CV12 (*n* = 9; *P < 0.05, **P < 0.01, and ***P < 0.001).

The modulatory effects of strong and slight MAS at ST36 or CV12 were investigated in the present study. As illustrated with an individual example in Figure 3(a) and with pooled data in Figures 3(b), 3(c), and 3(d), gastric motility was significantly facilitated by both strong and slight MAS at ST36 (amplitude changes: strong MAS: 46.9 ± 4.4%, *P* < 0.01; slight MAS: 23.5 ± 4.6%, *P* < 0.05) (integral changes: strong MAS: 50.4 ± 4.7%, *P* < 0.001; slight MAS: 34.5% ± 5.7%, *P* < 0.01) (frequency changes: strong MAS: 21.8 ± 4.9%, *P* < 0.01; slight MAS: 14.6 ± 4.6%, *P* < 0.05). On the contrary, as shown in Figure 4, strong and slight MA at CV12 produced significant inhibition on gastric motility (amplitude changes: strong MAS: −55.7 ± 8.2%, *P* < 0.01; slight MAS: −36.9 ± 6.4%, *P* < 0.01) (integral changes: strong MAS: −67.4 ± 3.4%, *P* < 0.001; slight MAS: −41.2% ± 1.5%, *P* < 0.01) (frequency changes: strong MAS: −51.2 ± 3.4%, *P* < 0.001; slight MAS: −36.3 ± 3.7%, *P* < 0.01). Notably, the modulatory effects induced by strong MAS with potent De-qi (needle grasp feeling) were markedly higher than those by slight MAS with mild De-qi sensation (*P* < 0.05, Figures 3(d) and 4(d)). In addition, the effectiveness of strong MAS in gastric motility was significantly higher than that of the last 60 s of 45°C LMS (*P* < 0.05, *P* < 0.01, and *P* < 0.001, Figure 5).

## 4. Discussion

In the present study, we investigated the possible correlation between intensity of stimulation, distinct afferent fibers, and generation of De-qi sensation. Through observation on the effects of nonnociceptive (41°C) and nociceptive (43°C, 45°C) LMS on gastric motility in rats, our results strongly indicated that both the facilitatory effect of ST36 and inhibitive effect of CV12 induced by LMS were closely related to the temperature intensity by which afferent fibers were activated. 43°C and 45°C LMS, rather than 41°C, produced significant regulatory effects on gastric motility. These results suggested that the nociceptive (>42°C) heat-activated A*δ*-/C- fibers were essential to the generation of De-qi and LMS-modulated gastric motility, whereas the nonnociceptive warm stimulus can hardly trigger De-qi sensation and the somatovisceral reflex. Additionally, the excitatory effect of ST36 and inhibitory effect of CV12 on gastric motility by MAS have also been demonstrated in this study. Notably, the regulatory effects of strong MAS were significantly higher than those of slight MAS, which might be contributed to the stronger De-qi sensation (needle grasp) induced by the former.

Increasing evidence showed that acupoints located in different parts of body produce different effects through specific somatoautonomic reflexes; for example, the facilitatory effect of ST36 at hindlimb on gastric motility was mediated via the parasympathetic pathway, whereas the inhibitory effect of acupuncture on abdomen was reasoned to be attributed to the sympathetic pathway [12, 13]. Using recording method of unitary discharge of nerve fibers, some studies showed that A*δ*- and C-afferent fibers were activated during MAS in humans and rats [14, 15]. Moreover, it is suggested that A-type fibers are activated when gentle MAS induces De-qi, whereas C-afferent fibers are involved in the enhancement of De-qi feeling when the needle is rotated and twisted repetitively [16]. It has been showed that rotation is the most commonly used manipulation for acupuncture to produce De-qi [17–19]. Consequently, it is conceivable that the De-qi feelings (needle grasp) elicited slight and strong MAS were more likely to be mediated by the activation of A*δ*- and C-afferent fibers.

The effects of moxibustion on various gastrointestinal diseases have been observed in humans and animals and it was showed that moxibustion has beneficial effects on improving gastrointestinal motility, protecting gastric mucosa, and relieving visceral hyperalgesia [20–22]. Temperature-related (local moxibustion-like stimulation, LMS) and nontemperature-related factors (smoke, odor, and herbs) are likely to be involved in the mechanism underlying the effectiveness of moxibustion [23]. Actually, the temperature was even emphasized and had been used as an alternative method of moxibustion in a lot of experimental studies [24, 25]. Previous studies proved that the mean heat-evoked responses of A*δ*- and C-afferent fibers were about 43°C [26–28]. Given the results in the present study, as a nociceptive heat, 43°C was essential to the effective regulation of gastric motility by LMS, which implied that A*δ*- and C-afferent fibers play critical roles in the generation of De-qi.

## 5. Conclusion

Taken together, the correlation between intensity of stimulation, afferent fibers, and the generation of De-qi feeling has been preliminarily demonstrated in the present study. Our results indicated that the generation of De-qi sensation induced by MAS and LMS was more likely to be mediated by A*δ*- and C-afferent fibers.
